# Advancing perioperative care with digital applications and wearables

**DOI:** 10.1038/s41746-025-01620-3

**Published:** 2025-04-19

**Authors:** Ben Li, Arjun Mahajan, Dylan Powell

**Affiliations:** 1 Division of Vascular Surgery, University of Toronto, Toronto, ON Canada; 2 Temerty Centre for Artificial Intelligence Research and Education in Medicine, University of Toronto, Toronto, ON Canada; 3 Harvard Medical School, Boston, MA USA; 4 Faculty of Health Sciences & Sport, University of Stirling, Stirling, UK

**Keywords:** Biomarkers, Health care

## Abstract

The rapid increase in real-time health information collected from wearable devices has allowed digital biomarkers to emerge as a promising tool to support perioperative care, including surgical prehabilitation, intra-operative guidance, and post-operative monitoring. Important challenges include the accuracy of generated information, data security risks, and slow adoption of new technologies. Active stakeholder engagement and following existing digital biomarker development/implementation frameworks may support using this technology to improve surgical outcomes.

## Introduction

**Fig. 1:**
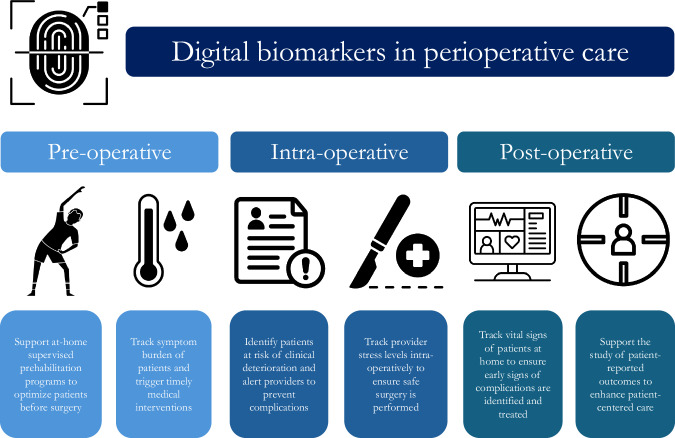
Potential applications of digital biomarkers in perioperative care.

Digital biomarkers are quantifiable measures collected from digital health technologies that may act as indicators of biological processes^1^. The rapid increase in real-time health information collected from wearable devices has allowed digital biomarkers to emerge as a promising tool to support the diagnosis, monitoring, and treatment of various health conditions^1^. While digital biomarkers have broad applications in health care, their utilization in perioperative medicine represents an emerging area of study, practice development, and implementation science^1^. A discussion of the potential applications of digital biomarkers in perioperative care may be informative to surgeons, anesthesiologists, and other clinicians involved in caring for patients undergoing surgery. This is particularly relevant given multiple recent publications on digital biomarkers in perioperative care^2–4^. In this article, we discuss the potential role of digital biomarkers in the pre-operative, in-hospital, and post-operative phases of care to improve patient outcomes (Fig. 1).

## Pre-operative care

A critical aspect of pre-operative care involves optimizing patients for surgery. Digital biomarkers can play an important role in prehabilitation, defined as interventions undertaken before surgery to reduce peri-operative risk^5^. Prehabilitation aims to improve patients’ functional capacity, nutritional status, and psychological readiness for surgery through exercise, dietary, and psychological support^6^. Digital biomarkers may support at-home prehabilitation programs, whereby patients can use wearable technologies to track their physical, nutritional, and mental health^7^. For example, Waller and colleagues (2022) showed that using wearable technologies during prehabilitation increased patients’ physical activity, walking distance, and functional status^8^. Notably, multiple mobile applications have been developed to support surgical prehabilitation^9–12^. Another central aspect of pre-operative care involves managing symptom burden^13^. At clinic visits, it may be challenging to completely appreciate the impact of illness on patients’ lives^14^. An example of the utility of digital biomarkers in pre-operative care is illustrated by Low and colleagues (2021), who showed that digital biomarker data captured through smartphones and smartwatches at home could be used to track patient-reported symptom burden based on activity patterns, sleep, screen time, pain, and fatigue in individuals scheduled for pancreatic surgery^15^. This may help patients and providers detect worsening perioperative symptoms and trigger timely symptom management interventions^15^.

## In-hospital care

While in hospital, clinicians are often responsible for caring for patients with different acuity levels^16^. Digital biomarker systems using artificial intelligence may rapidly synthesize health indicators in real time, notifying providers promptly when patients show early signs of decompensation^17^. For example, the CHARTWatch system continuously monitors over 100 clinical variables for hospitalized patients and predicts their risk of requiring intensive care unit care or detioration within the next 48 hours. It provides a warning notification to the medical team for these high-risk patients so they can receive appropriate care^18^. Preliminary data demonstrated that the algorithm predicted patient outcomes >15% more accurately than clinicians, resulting in a ~15% reduction in mortality among high-risk patients^18^. This may be particularly helpful in surgery due to the high proportion of patients who could rapidly deteriorate given their medical complexity and often high-risk procedures^19^. Digital biomarker information may also be helpful for clinicians intra-operatively by measuring their stress levels. For example, Dias and colleagues (2023) used digital biomarker data from wearable sensors for heart rate variability that accurately captured acute stress levels in perfusionists operating the heart-lung machine during cardiac surgery^4^. Given that extremely high stress levels can lead to major intraoperative complications, a timely alert regarding a clinician’s level of stress can prompt the operative team to consider slowing down, re-evaluating the situation, and ensuring that safe surgery is provided^20^.

## Post-operative care

**Fig. 2:**
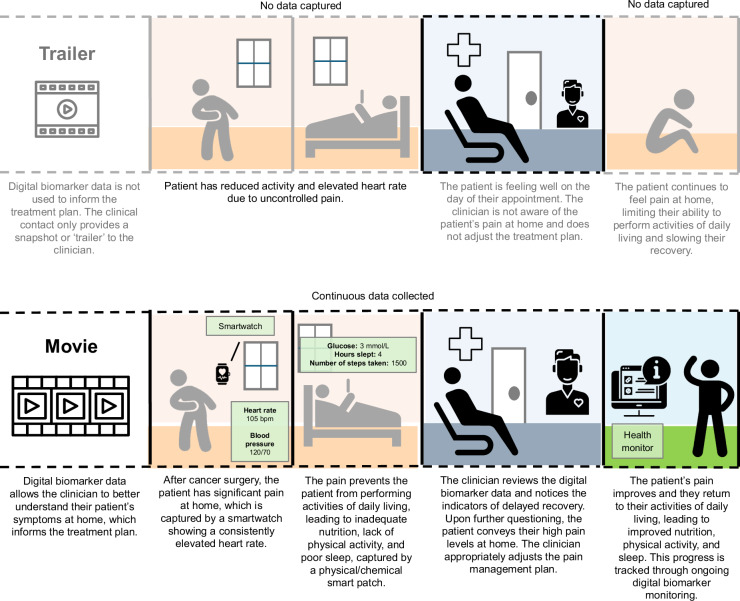
‘Trailer versus the Movie’: An example of how digital biomarkers can improve a cancer patient’s perioperative care journey.

After surgery, patients generally stay in hospital for several days or weeks until clinicians deem them to be fit to go home. However, most post-operative recovery occurs at home, particularly after major operations. During this transition, patients can often abruptly go from having every heartbeat monitored to minimal vital sign tracking. As a result, a significant proportion of patients are at risk of postoperative complications after they are discharged^21^. Digital biomarker systems informed by wearable technologies can allow patients and their providers to track important vital signs at home^22^. When there are signs of decompensation, an alert may be sent to the provider and/or patient to ensure that the patient seeks appropriate care^22^. Additionally, the study of patient-reported outcomes has become a priority in surgery^23^. One significant challenge is the current inability to capture longitudinal patient-reported information reliably^23^. Wearable technologies and digital biomarkers can allow patients to track their recovery process at home effectively and report outcomes most relevant to their well-being in a feasible and cost-effective manner^23^. For example, Kolk and colleagues (2023) showed that digital biomarker information obtained from wearable devices strongly correlated with patient-reported outcome measures in patients with implantable cardioverter defibrillators^24^. An example of how digital biomarkers may be used to better capture longitudinal patient-related information that can improve a clinician’s understanding of their patients and guide treatment plans is illustrated in Fig. 2.

## Challenges

While digital biomarkers have important potential to improve perioperative care, several challenges must be considered. First, wearable products and digital technologies may generate inaccurate data. This is particularly concerning if these errors exacerbate structural health inequalities. For example, Sjoding and colleagues (2020) demonstrated that pulse oximeters had increased error rates in patients with different skin pigmentation^25^. Therefore, digital biomarkers must be validated on diverse and representative datasets before implementation. Second, digital biomarker data in health care should be confidential and shared only with the patient’s permission. Given the known potential for cyber threats to compromise the security of personal health information, data privacy must be a central consideration when designing digital biomarker systems^26^. Third, medicine can be slow to adopt new technologies, given their potential to have unintended negative consequences^27^. Therefore, co-development involving patients, clinicians, and healthcare administrators in designing and implementing digital biomarkers is critical. Conscientious stakeholder engagement will build trust, demonstrate transparency, and ultimately facilitate safe uptake of this technology.

## Conclusions and next steps

Digital biomarkers derived from increasingly available wearable and digital technologies have the potential to augment and improve pre-operative, in-hospital, and post-operative care. This tool may support prehabilitation, track symptom burden, identify early signs of clinical deterioration, and capture patient-reported outcomes, among other applications. Although challenges of data accuracy, patient privacy, and slow uptake of new technologies in health care exist, active stakeholder engagement and collaboration may allow for the development of effective digital biomarkers that can improve surgical outcomes. By following existing frameworks such as the one developed by Daniore and colleagues^28^, considering issues of data collection, aggregation, contextualization, interpretation, and action, wearable sensor data can be effectively translated to digital biomarkers that can transform perioperative care.

## Data Availability

No datasets were generated or analysed during the current study.
